# Direct evidence of active SARS-CoV-2 replication in the intestine

**DOI:** 10.1093/cid/ciaa925

**Published:** 2020-07-08

**Authors:** Qun Qian, Lifang Fan, Weicheng Liu, Jin Li, Junqiu Yue, Mingwei Wang, Xianliang Ke, Yan Yin, Quanjiao Chen, Congqing Jiang

**Affiliations:** 1 Department of Colorectal and Anal Surgery, Zhongnan Hospital of Wuhan University, Wuhan, China; 2 Clinical Center of Intestinal and Colorectal Diseases of Hubei Province, Wuhan, China; 3 Hubei Key Laboratory of Intestinal and Colorectal Diseases (Zhongnan Hospital of Wuhan University), Wuhan, China; 4 Colorectal and Anal Disease Research Center of Medical School (Zhongnan Hospital of Wuhan University), Wuhan, China; 5 Quality Control Center of Colorectal and Anal Surgery of Health Commission of Hubei Province, Wuhan, China; 6 Department of pathology, Hubei Cancer Hospital, Wuhan, China; 7 Department of Laboratory Medicine, Zhongnan Hospital of Wuhan University, Wuhan, China; 8 CAS Key Laboratory of Special Pathogens and Biosafety, Wuhan Institute of Virology, Center for Biosafety Mega-Science, CAS Center for Influenza Research and Early Warning, Chinese Academy of Sciences, Wuhan, China

**Keywords:** SARS-CoV-2, Coronavirus disease 2019 (COVID-19), intestinal infection rectal cancer

## Abstract

**Background:**

Currently, there is no direct evidence to prove the active SARS-CoV-2 replication in the intestinal tract and relevant pathological changes in the colon and rectum. We investigated the presence of virions and pathological changes in surgical rectal tissues of a clinically confirmed COVID-19 patient with rectal adenocarcinoma.

**Methods:**

Here, the clinical data were collected during hospitalization and follow-up of this patient. Quantitative RT-PCR was performed on the rectal tissue specimens obtained from surgical resection, succus entericus and intestinal mucosa of ileostomy, and rectal mucosa during follow-up after recovery. Ultrathin sections of surgical samples were observed for SARS-CoV-2 virions using electron microscopy. Histopathological examination was performed using hematoxylin-eosin stain. Immunohistochemical analysis and immunofluorescence were carried out on rectal tissues to evaluate the distribution of SARS-CoV-2 antigen, and immune cell infiltrations.

**Results:**

The patient had fever and cough on day 3 postoperatively, was diagnosed with COVID-19 on day 7, and was discharged from the hospital on day 41. RNA of SARS-CoV-2 was detected in surgically resected rectal specimens, but not in samples collected on 37 day after discharge. Notably, coincidence with rectal tissues of surgical specimens tested nucleic acid positive for SARS-CoV-2, typical coronavirus virions in rectal tissue were observed under electron microscopy. Moreover, abundant lymphocytes and macrophages (some are SARS-CoV-2 positive) infiltrating the lamina propria were found with no significant mucosal damage.

**Conclusions:**

We firstly reported that direct evidence of the active SARS-CoV-2 replication in the patient's rectum during the incubation period, which might explain SARS-CoV-2 fecal-oral transmission.

found with no significant mucosal damage.

**Conclusions**

We firstly reported that direct evidence of the active SARS-CoV-2 replication in the patient's rectum during the incubation period, which might explain SARS-CoV-2 fecal-oral transmission.

## INTRODUCTION

The current coronavirus disease identified in 2019 (COVID-19), caused by a novel coronavirus, has become a global public health problem [1, 2]. As of May 27, 2020, a total of 5,451,532 cases of COVID-19 have been confirmed globally, including 345,752 deaths [3]. There are many reports suggesting that SARS-CoV-2 RNA can be detected and identified in anal/rectal swabs [4, 5] and stool specimens [6, 7]. In fact, one recent small sample study found that RNA was consistently detected in rectal swabs, even after viral clearance from the upper respiratory tract, indicating extended duration of viral shedding in fecal samples and raising the possibility of fecal-oral transmission of SARS-CoV-2 [5]. Similar results were reported in another study with more cases involved, raising the possibility of prolonged presence of SARS-CoV-2 in stools. Notably, fecal samples remained positive for SARS-CoV-2 RNA nearly 5 weeks after the viral clearance from the upper respiratory tract in COVID-19 patients [8]. Considering a high degree of sequence homology between the SARS-CoV-2 and SARS-CoV, angiotensin-converting enzyme II (ACE2) has been identified as the entry receptor of SARS-CoV-2. Since this receptor is highly expressed on the epithelial cells from the ileum and colon [9], the intestinal tract may be a potential route for SARA-CoV-2 infection and transmission.

Patients with cancer are considered to be more susceptible to SARS-CoV-2 [10, 11]. One patient with rectal cancer was admitted to Zhongnan Hospital of Wuhan University for radical operation. On postoperative day 3, the patient began to develop cough and fever; chest CT revealed radiologic characteristics of viral pneumonia. On postoperative day 7, the patient was confirmed to be infected with SARS-CoV-2. Although live SARS-CoV-2 had been successfully isolated from the fecal sample of a laboratory confirmed SARS-CoV-2 patient [12], until now, there has been no direct evidence to prove active SARS-CoV-2 virus replication in the intestinal tract. It remains unknown whether there are pathological changes related to SARS-CoV-2 infection existing in colorectal mucosa in COVID-19 patients. To clarify the above questions, we performed a retrospective study to detect the presence of SARS-CoV-2 virions and determine the pathological changes in rectal tissues of this patient.

## METHODS

### Patients and associated procedures

The patient's clinical information is described in Supplementary Table 1. The small pieces of stored rectal tissues obtained from surgical specimens during the operation on January 16, 2020, were used for retrospective detection. This study was approved by the Ethics Committee of Chinese Clinical Trial Registry (reference number: ChiECRCT20200116).

### Real-time RT-PCR

Samples of rectal tissues, succus entericus and intestinal mucosa of ileostomy, and rectal mucosa were tested for SARS-CoV-2 nucleic acid using qRT-PCR. The qRT-PCR analyses were performed following a previously described method [13]. The qRT-PCR test kits (BioGerm) were recommended by the Chinese Center for Disease Control and Prevention.

**Electron microscopy**

The rectal tissue obtained by resection was soaked in RNAlater solution overnight, the solution was discarded and the tissue was frozen at -80°C. The rectal tissue was cut into 1 mm thick sections and fixed in 2.5% glutaraldehyde and 1% osmium tetroxide in a biosafety cabinet with level 2 protection, and subsequently dehydrated using different ascending concentrations of alcohol (30% to 100%), and immersed and embedded in epoxy resin. Ultrathin sections (80-100 nm) were prepared on formvar-coated copper grids (200 mesh). The virions were observed with a Tecnai G2 20 TWIN electron microscope (FEI company) under 200 kV.

**Immunochemistry and immunofluorescence assay**

Immunohistochemical staining was performed on formalin-fixed and paraffin-embedded tissue sections (4-μm). Sections of rectal tissues were immunostained to evaluate the expression and distribution of the SARS-CoV-2 antigen. Briefly, sections were deparaffinized with xylene and alcohol and subsequently heated in citrate buffer (pH 6.0) for antigen retrieval. After blockage with 3% BSA in PBS for 30 minutes, a rabbit antibody against Rp3 NP protein (kindly provided by Dr. Zhengli Shi, Wuhan Institute of Virology, Chinese Academy of Science [14]) was incubated with the sections overnight at 4°C. For immunochemistry study, the slides were subsequently incubated with HRP-conjugated goat anti-rabbit IgG (Promoter Biotech, China) for 1 hour at 37°C. Then sections were stained with DAB and hematoxylin. For immunofluorescence assay, Cy3-conjugated goat anti-rabbit IgG (Abcam, USA) was used as a secondary antibody. Images were acquired using a Panoramic scanner (3D-Histech, Hungary) or a fluorescence microscope (Olympus IX51). CD117(rabbit polyclonal anti-human) and CD20(L26) were stained on the Leica Bond-Max autostainer (Lecia Microsystems, Chicago, IL), and CD3(P7.2.28), CD4(4B12), CD5(4C7), CD8(C814423), CD38(SP149) and CD68(KP1) were stained on DAKO Autostainer Link48.

**RESULTS**

Timeline of SARS-CoV-2 infection after rectal cancer surgery is shown in Figure 1. The patient's clinical information is shown in supplementary Table 1. On January 16, 2020, the patient underwent rectal surgery. The surgically removed tissue was used for pathological diagnosis. A small portion of the remaining tissue was soaked with RNA later solution for 24 hours and transferred to a $-80^{\circ}$ C freezer. On day 3 postoperatively, the patient presented with fever and cough. On day 7 postoperatively, chest CT scan showed typical viral pneumonia with ground-glass opacity (Figure 2). At the same time, the SARS-CoV-2 infection was confirmed by real-time reverse transcription–polymerase chain reaction (qRT-PCR) assay using throat swab samples and the patient was transferred to an isolation ward for treatment. The patient was discharged on day 41 after two consecutive negative qRT-PCR test results plus absence of clinical symptoms and radiological abnormalities. In the middle of March, we conducted a retrospective study on the patient's surgically removed tissue. Firstly, SARS-CoV-2 nucleic acid detection was performed on the surgical specimens of rectal tissues, which was positive for SARS-CoV-2. In addition, throat swab, rectal swab, terminal ileum mucosa, and succus entericus samples were collected on day 72 during follow-up and tested for SARS-CoV-2 nucleic acid; all of these were found to be negative for SARS-CoV-2.

To explore the direct evidence of SARS-CoV-2 infection and replication in the rectal tissues of surgical specimens, ultrathin sections of rectal tissues were prepared, and virus particles were found in the cytoplasm of intestinal epithelial cells. Under electron microscopy, the virions showed typical morphology of coronavirus (Figure 3).

Pathological changes of rectal mucosa with SARS-CoV-2 infection and replication are shown in Figure 4. Hematoxylin and eosin-stained rectal mucosa showed prominent lymphocytes and macrophages infiltrating the lamina propria without significant mucosal damage (Figure 5). T lymphocytes and macrophages were found to be more numerous than B lymphocytes in the lamina propria, as demonstrated on immunostaining. No viral inclusions were observed in the tissues.

To further confirm the SARS-CoV-2 -specific infection and replication in the rectum, we conducted immunohistochemistry and immunofluorescence using the rabbit anti-SARA-CoV-2 NP antibody. SARS-CoV-2 antigens were confirmed to be expressed on intestinal epithelial cells, lymphocytes and macrophages in the lamina propria (Figure 6).

## DISCUSSION

In this study, we found, for the first time, that the SARS-CoV-2 had already replicated in the patient's rectum during the incubation period, with no obvious intestinal pathological damage. In this case, the patient developed a dry cough and fever (39.2℃) in the early stage after the operation (postoperative day 3). At the same time, an atypical pulmonary inflammation was revealed by the chest CT. Fever and pulmonary infection are common complications after surgery. However, numerous possible causes of these clinical manifestations to some extent led to a challenge in the differential diagnosis between SARS-CoV-2-related fever and postoperative fever. Initially, when the patient started developing fever, the throat swab was not tested for SARS-CoV-2.

Here, we observed typical SARS-CoV-2 virus particles in the intestinal epithelial cells of patients under electron microscopy and obtained direct evidence of active SARS-CoV-2 virus replication in the intestine. Meanwhile, we detected the viral components of SARS-CoV-2 in the intestine. It is worth mentioning that virus particles were found in intestinal epithelial cells, but the results of immunofluorescence and immunohistochemistry showed that the viral components were mainly present in intestinal lymphocytes and macrophages, besides the intestinal epithelial cells. To explain this seemingly self-contradictory phenomenon, we need to understand the scientific question of how the virus spreads from the lungs to other non-pulmonary tissues. Regarding this issue, some researches have been carried out in the influenza virus and several studies have shown that influenza viruses can be transported by immune cells and spread from the lungs to other tissues [15-17]. These "virus-carrying”

immune cells contain professional antigen-presenting cells such as macrophage, monocytes, and dendritic cells [18-21]. Influenza virus RNA carried by immune cells includes viral RNA fragments (majority, these cannot infect other cells) and whole RNA virions (minority, these can infect other cells). These are jointly called the influenza virus transcriptome. Several researches have strongly supported the concept that the influenza virus transcriptome can be transported by the immune cells within the systematic circulation, following which, the viral transcriptome could spread to distant organs such as the intestine[21]. We speculate that the SARS-CoV-2 virus uses the same strategy as the influenza virus to spread from the lung to the distant organs. Therefore, a large number of non-infectious components of SARS-CoV-2 in intestinal lymphocytes and macrophages were found, with few components in the intestinal epithelial cells. Of course, further experiments are needed to verify this hypothesis.

Our study provided further evidence to support the fecal-oral transmission route of SARS-CoV-2, and the intestine represent a target organ of SARS-CoV-2 [22]. A recent study indicated persistent presence of SARS-CoV-2 RNA in stool samples of SARS-CoV-2-positive patients with moderate disease, even 5 weeks after onset of symptoms[8]. However, in this study, no SARS-CoV-2 RNA was detected in samples from the gastrointestinal tract in this case on day 37 after hospital discharge. To explore the mechanism of high viral load and persistence of SARS-CoV-2 RNA in the intestinal tract of some patients when SARS- CoV- 2 nucleic acid tests of throat swab was negative and clinical indicators were normal, further studies with large sample size are needed.

**Acknowledgements**

We are grateful to Center for Instrumental Analysis and Metrology, The Core Facility and Technical Support, Wuhan Institute of Virology, CAS.

**Funding**

This work was supported by the National Science and Technology Major Projects [grant number 2020YFC0846200].

**Conflict of interest**

We declare that we have no conflicts of interest.

**Figure legends**

**Figure 1. Timeline of SARS-CoV-2 infection after rectal cancer surgery.**

**Figure 2. The chest CT of the patient.** (A) On day 2 before the operation. No abnormalities in the lung. (B) On day 3 after the operation. Infection was found in the bilateral lower lung fields but without radiologic characteristics of the SARS-CoV-2 infection. (C) On day 7 after the operation. The blue arrow shows the ground glass opacities affecting bilateral, subpleural lung parenchyma, which are radiologic characteristics of the SARS-CoV-2 infection. (D) On day 14 after the operation. The blue arrow shows the ground glass opacities, and the consolidation dissipated into irregular linear fibrosis and irregular fibrosis foci.

**Figure 3. SARS-CoV-2 Virions in the intestinal tissue.** The intestinal tissue was used to prepare ultrathin sections. The viral particles were observed under electron microscope at 200 kV.

**Figure 4. Pathological changes of the rectal mucosa.** The abundant lymphoplasma cells in the lamina propria with intact mucosal architecture.

**Figure 5. H&E staining and immunostaining of rectal mucosa.** The same field in the tissue to Figure 4. (A) H&E staining, abundant lymphoplasma cells in the lamina propria. (B) CD4-positive T cells in the lamina propria. (C) CD8-positive T cells in the epithelium and the lamina propria. (D) CD68-positive macrophages in the lamina propria. (E) CD3-positive T cells in the epithelium and the lamina propria. (F) CD5-positive T cells in the epithelium and the lamina propria. (G) CD38-positive plasma cell. (H) Minimal CD20-positive B cells in the lamina propria. (I) Minimal CD117-

positive mast cells in the lamina propria.

**Figure 6. The SARS-CoV-2 infection in the intestinal tissue**. (A) (B) Immunochemistry image with the SARS-CoV-2 antigen. (C) Immunofluorescence image with the SARS-CoV-2 antigen.

**Figure 1**

**Figure 2**

**Figure 3**

**Figure 4**

**Figure 5**
