# Supplementary material for: Direct Evidence of Active SARS-CoV-2 Replication in the Intestine
Source: Clin Infect Dis. 2020 Jul 8;73(3):361–6. doi: 10.1093/cid/ciaa925 (PMC7454471; doi:10.1093/cid/ciaa925)
Supplement: ciaa925_suppl_Supplementary_Table_1 [file ciaa925_suppl_supplementary_table_1.doc]

| **Measure** | **Reference**  **Range** | **Baseline** | **Operation** |  | **Fever and cough** | | | |  |  |  |  |  |  | **Follow-up** |
| --- | --- | --- | --- | --- | --- | --- | --- | --- | --- | --- | --- | --- | --- | --- | --- |
| **Day of operation** |  | **-3** | **0** | **2** | **3** | **4** | **5** | **7** | **13** | **20** | **24** | **25** | **28** | **36** |  |
| WBC (×10^9^/L) | 3.5--9.5 | 4.86 | 6.70 | 7.60 | 6.90 | **9.72 ↑** | 8.28 | 3.85 | 4.44 | **2.70 ↓** | 4.04 | 4.38 | 5.2 | 4.9 | 5.61 |
| LYM (×10^9^/L) | 1.1--3.2 | **0.57 ↓** | **0.27 ↓** | **0.61 ↓** | **0.29 ↓** | **0.69 ↓** | **0.66 ↓** | **0.44 ↓** | **1.08 ↓** | **0.88 ↓** | **1.07 ↓** | 1.19 | 1.72 | 1.48 | 1.33 |
| LYM (%) | 20--50 | **11.8 ↓** | **4.0 ↓** | **8.0 ↓** | **4.2 ↓** | **7.10 ↓** | **8.0 ↓** | **11.4 ↓** | 24.3 | 32.8 | 26.5 | 27.1 | 33.3 | 29.9 | 23.7 |
| NEUT(×10^9^/L) | 1.8--6.3 | 3.80 | 6.24 | **6.50 ↑** | **6.35 ↑** | **8.38 ↑** | **6.89 ↑** | 2.74 | 2.63 | **1.21 ↓** | 2.22 | 2.43 | 2.59 | 2.64 | 3.75 |
| NEUT (%) | 40--75 | **78.3 ↑** | **93.2 ↑** | **85.5 ↑** | **92.0 ↑** | **86.20 ↑** | **83.2 ↑** | 71.0 | 59.3 | 45.0 | 55.1 | 55.5 | 49.9 | 53.5 | 66.9 |
| PLT (×10^9^/L) | 125--350 | 180 | 137 | **115 ↓** | **124 ↓** | 159.00 | 170 | **107.0 ↓** | **421 ↑** | 280 | 151 | 134 | 123 |  | 190 |
| IL-6（pg/mL） | 0--7 |  | **522.2 ↑** |  |  |  |  |  | 6.44 | 2.42 |  |  |  |  |  |
